# Modeling the airborne survival of influenza virus in a residential setting: the impacts of home humidification

**DOI:** 10.1186/1476-069X-9-55

**Published:** 2010-09-03

**Authors:** Theodore A Myatt, Matthew H Kaufman, Joseph G Allen, David L MacIntosh, M Patricia Fabian, James J McDevitt

**Affiliations:** 1 Environmental Health & Engineering, Inc., Needham, MA, USA; 2 Harvard School of Public Health, Department of Environmental Health, Boston, MA, USA

## Abstract

**Background:**

Laboratory research studies indicate that aerosolized influenza viruses survive for longer periods at low relative humidity (RH) conditions. Further analysis has shown that absolute humidity (AH) may be an improved predictor of virus survival in the environment. Maintaining airborne moisture levels that reduce survival of the virus in the air and on surfaces could be another tool for managing public health risks of influenza.

**Methods:**

A multi-zone indoor air quality model was used to evaluate the ability of portable humidifiers to control moisture content of the air and the potential related benefit of decreasing survival of influenza viruses in single-family residences. We modeled indoor AH and influenza virus concentrations during winter months (Northeast US) using the CONTAM multi-zone indoor air quality model. A two-story residential template was used under two different ventilation conditions - forced hot air and radiant heating. Humidity was evaluated on a room-specific and whole house basis. Estimates of emission rates for influenza virus were particle-size specific and derived from published studies and included emissions during both tidal breathing and coughing events. The survival of the influenza virus was determined based on the established relationship between AH and virus survival.

**Results:**

The presence of a portable humidifier with an output of 0.16 kg water per hour in the bedroom resulted in an increase in median sleeping hours AH/RH levels of 11 to 19% compared to periods without a humidifier present. The associated percent decrease in influenza virus survival was 17.5 - 31.6%. Distribution of water vapor through a residence was estimated to yield 3 to 12% increases in AH/RH and 7.8-13.9% reductions in influenza virus survival.

**Conclusion:**

This modeling analysis demonstrates the potential benefit of portable residential humidifiers in reducing the survival of aerosolized influenza virus by controlling humidity indoors.

## Background

Annual influenza epidemics exhibit a strong seasonal cycle in temperate regions. Due to the cyclical nature, it has long been assumed that environmental factors played a role in the seasonal epidemics [1]. Numerous laboratory studies have demonstrated that aerosolized influenza virus survival in the air and on surfaces is affected by temperature and more importantly, relative humidity (RH) [2-9]. These studies, carried out using a variety of methods, show that aerosolized influenza virus survives substantially longer at low RH levels. For example, the results of the study conducted by Harper, show that aerosolized influenza survived best when the RH was below 36%, with a sudden decrease in survival of the virus when the RH was raised above 49% [7]. While the data are more limited, studies have also shown RH impacts influenza virus survival on surfaces [10-12].

RH, the ratio of the vapor pressure of water to the saturation vapor pressure at a prescribed temperature and pressure, has been the parameter of interest in past influenza survival and transmission studies. In a reanalysis of influenza survival and transmission data Shaman and Kohn show that compared to RH, absolute humidity (AH), the mass of water per volume of air, has a much stronger statistically significant relationship with influenza virus survival [13]. These results have been extended in an epidemiological model that indicates that AH, as a modulator of influenza transmission, drives seasonal variations of influenza transmission in temperate regions [14].

Indoor moisture levels are dependent on outdoor moisture loads, indoor moisture sources and ventilation rates. Studies conducted in Finland, Canada and Wisconsin have shown that heating season indoor RH levels are low, ranging between 15 and 45% with mean levels of approximately 35% [15-17] which corresponds to 8.1 millibar (mb) AH (at 20°C and standard pressure). The laboratory studies discussed above suggest that increasing the moisture levels above typical indoor AH and RH may mitigate the spread of influenza viruses in the air and on surfaces during the influenza season [13,18].

The objective of this study was to evaluate the effect of using portable humidifiers on AH levels and influenza virus survival in Northeast United States residences using the CONTAM multi-zone indoor air quality model.

## Methods

The CONTAM multi-zone indoor air quality model (National Institute of Standards and Technology (NIST), Gaithersburg, MD), was used to estimate moisture levels (i.e., RH and AH) and indoor influenza virus concentrations indoors [19]. CONTAM generates dynamic simulations of inter-zonal airflows, ventilation rates, and concentrations of gaseous and aerosol contaminants. The performance of the model has been evaluated extensively. Estimated inter-zonal airflows and air exchange rates have been shown to be within 15% on average of actual measurements and modeled fine particle levels within 30% of measured values [20-23].

Our analysis utilized a two-story 88 m^3 ^detached residential building template developed by NIST (Template DH-28). Simulations were conducted for two types of heating systems: a forced hot air system and a radiant heating system. The primary difference between the two systems for purposes of this analysis is that forced air systems provide more rapid mixing of indoor air throughout a residence than radiant heating. The forced air system provided 0.18 m^3^/min/m^2 ^of air to each room in the house. The air handler duty schedule was simulated with 1 hour resolution based on output from the EnergyPlus Energy Simulation Software [24]. In general, the fraction of each hour devoted to forced air heating was proportional to the difference between ambient temperature and a set point of 17.8°C (64°F) from 10 PM to 5 AM and 22.2°C (72°F) from 5 AM to 10 PM. Additional details of the residential template and heating systems are described elsewhere [25,26].

Indoor moisture sources and their generation rates were based on published data [27]. Indoor moisture sources included cooking, dishwashing, bathing, and both waking and sleeping occupants. After inclusion of these sources, the model was calibrated to achieve average indoor RH levels reported for homes during the heating seasons [15-17]. A humidifier moisture source with a generation of 0.16 kg/hr (Model V4500, Kaz, Inc., Southborough, MA) was added to either a single bedroom or all the bedrooms and the family room depending on the modeling scenario.

Meteorological information is used by CONTAM to simulate force convection, radiant leakage, and corresponding air exchange rates. We used National Renewable Energy Laboratory TMY2 typical meteorological year meteorological data during the Northern hemisphere influenza season (October to March), including hourly wind direction and speed, dry and wet bulb temperature, relative humidity, and cloud cover data, obtained from the National Weather Service for Boston, Massachusetts (WBAN 14739 Boston Logan International Airport). The Boston area was chosen because, similar to other areas of the Northern US, Boston experiences long periods of cold, dry weather during the winter months and is therefore likely to have low indoor moisture levels.

Our modeling assumed a single influenza case in the bedroom and evaluated the levels of the virus in the bedroom and outside the bedroom. Emission rates for influenza virus were derived from our previous studies with regard to particle counts, particle sizes, and influenza virus RNA concentrations in persons with confirmed flu and were used as input into the CONTAM model [28-30]. In these previous studies, tidal breathing, particle counts and influenza virus RNA concentrations were measured using an Exhalair device (Pulmatrix, Lexington, MA), which records particle counts between 0.3 um and >5 um with an optical particle counter, and collects exhaled breath particles on Teflon filters. In our prior research for coughs, influenza virus RNA was collected with the Gesundheit II device [31], which collects particles by impaction in two size fractions: fine (< 5 μm) and coarse (> 5 μm). Influenza virus RNA was measured in these previous studies by a reverse transcription-quantitative polymerase chain reaction (RT-qPCR) assay. The relationship of 300 viral RNA copies per infective virus particle (determined via cell culture assays), determined in laboratory studies, was used to convert the concentration of influenza virus RNA copies in each particle to infective virus particles per particle size bin [30].

We modeled tidal breathing as a constant emission source of influenza particles per minute in four different particle sizes. Coughing emissions were modeled as one second bursts of infective influenza particles in fine and coarse particle sizes. A frequency of 15 coughing episodes per hour was based on experimental data of subjects with respiratory illness [32]. Influenza emissions were limited to the evening hours (7 PM to 10 AM) as the evening was assumed to be the period in which a person was most likely to be in a bedroom and most likely to be operating a portable humidifier. Emission rate details are presented in Table 1.

**Table 1:** Infective Influenza Virus Emission Rates

Source type	Particle size (μm)	Infectious influenza viruses per second	Source description
Cough	2.5	0.62	15 one second episodes per hour during sleeping hours
		
	7.5	0.11	

Tidal breathing	0.4	8.8E-05	Constant emission during sleeping hours
		
	0.75	1.9E-05	
		
	2.5	3.3E-06	
		
	7.5	7.8E-07	

To estimate the decrease of influenza virus in the air (i.e., biological decay) due to the increase in AH, the modeled airborne influenza virus levels, which accounts for the physical decay of the viral particles, were adjusted based on laboratory test data originally published by Harper and reanalyzed by Shaman and Kohn which exhibited a strong statistical relationship between AH and one hour loss of live virus [7,13]. Briefly, the adjustment was based on the regression of log (percent surviving after one hour) and AH (p-value< 0.0001) presented as Figure three, part F in Shaman and Kohn (2009). Presentations of results with RH assume an indoor temperature of 17.8°C (64°F).

## Results

In the models without humidifiers, the median night hour indoor bedroom moisture level was 33% RH (range: 12 to 65% RH) and 35% RH (range: 7 to 75% RH) for the radiant heat and forced air heat models, respectively. The RH levels translate into median AH levels of 7.5 mb for the radiant heat model and 8.2 mb for the forced air heat model.

**Figure 1:**
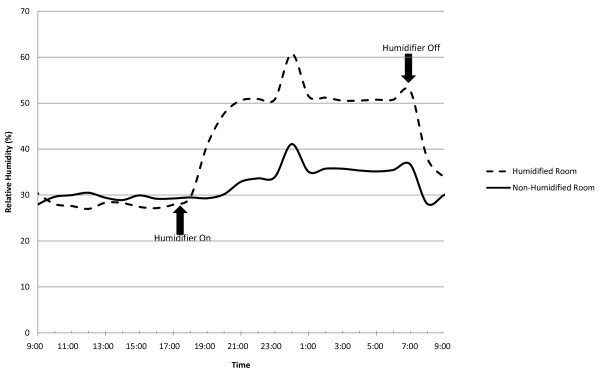
**Impact of humidifier on typical day (November 14 to November 15) on relative humidity in bedroom with humidifier operating from 6 PM to 7 AM**.

The addition of a humidifier in one bedroom increased the median night hour bedroom moisture level to 47% RH (median AH: 10.4 mb) for the radiant heat model and 41% (median AH: 9.4 mb) for the forced air heat model. The effect of the single humidifier on RH levels for a typical 24-hour period (November 14 to 15) is depicted in Figure 1. Prior to activating the humidifier, both models show room RH to be approximately 30%. After the humidifier is turned on (6 pm), RH levels increase to 40-60% RH (approximately 15-20% above baseline) and remain elevated for the duration of humidifier operation. The spike in RH depicted in the figure is due to an outdoor air event, and not related to an increase in moisture generated indoors. In the scenario with humidifiers in the four bedrooms and the family room, the whole house median moisture level increased to approximately 42% (AH: 10.0 mb) for both the radiant and forced air heat models.

**Figure 2:**
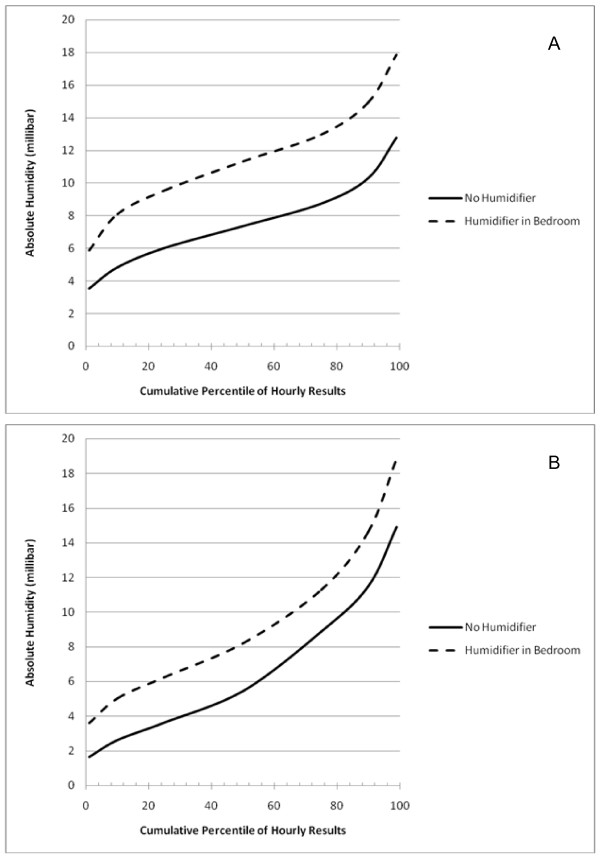
**Cumulative percentiles of hourly AH comparing rooms with and without humidifiers in the single humidifier in bedroom scenario in (A) model with radiant heat and (B) model with forced-air heat**.

Cumulative distributions of modeled hourly bedroom indoor AH levels over the entire modeling period (i.e., October to March) are shown in Figures 2A and 2B. For radiant heat, humidification increased the AH approximately 4 mb, while the increase was approximately one-half as large for the forced air heat.

The benefit of decreased influenza virus survival due to humidification for the single and multiple humidifier scenarios are presented in Table 2. Estimates of the change in influenza virus survival ranged from 17.5% to 31.6% reduction in rooms with a humidifier operating. The largest decrease in influenza virus survival was in the home with radiant heat due to the larger increase in moisture levels in the room. When multiple humidifiers are employed, the rooms with humidifiers have similar reductions to the single humidifier scenario, but the decrease in virus survival on the entire first and second floors are more modest.

**Table 2:** Decrease in Influenza Virus Survival due to Humidification, based on CONTAM Output

Heating Type	Modeled Domain^1^	No Humidification		Humidification		Decrease in Airborne Virus Survival due to Humidification (% change)
			
		Median hourly RH^2^	Median hourly AH (VP, mb)^2^	Median hourly RH^2^	Median hourly AH (VP, mb)^2^	
Bedroom Humidifier Scenario						

Forced Air	bedroom	27	5.4	38	7.7	17.5
		
Radiant		34	6.9	53	10.7	31.6

Multiple Humidifier Scenario^3^						

Forced Air	1st floor	26	5.2	29	5.8	7.8

	2nd floor	27	5.4	33	6.6	8

Radiant	1st floor	33	6.6	40	8.1	9.5
	
	2nd floor	36	7.3	48	9.7	13.9

^1 ^1st floor and 2nd floor are floor level averages

^2 ^Evening hour median levels (7 PM to 10 AM)

^3 ^Humidifiers in all bedrooms and family room

While the reductions in influenza virus survival due to humidification were greater for the radiant heat model, the concentration of viruses surviving in the bedroom was 1.9 fold greater in this model compared to the forced air model. The difference in concentration is due to greater air movement between rooms in the forced air model. Virus concentrations outside the bedroom was substantially lower than in the bedroom, with viral concentrations 27 and 77 fold lower in the hallway compared to the bedroom for the forced air and radiant heat models, respectively.

## Discussion

Results from this modeling analysis demonstrate that the use of portable residential humidifiers increases RH and AH to levels that can potentially decrease the survival of airborne influenza virus in a residential setting. This effect is more pronounced in rooms where the humidification is located. While this study evaluated the impacts in a residential setting, the expected benefits of humidification are likely to be larger in places where larger populations of people with the flu and people susceptible to the flu congregate.

Increasing low indoor moisture levels may have benefits beyond reducing survival of the influenza virus. Low RH has been associated with a number of symptoms including dry skin, throat and mucous membranes and eye irritation in office and hospital workers [33-35]. In a home humidification intervention, the authors reported a decrease in dryness of the nose and throat and improved breathing in patients with allergies [36].

While there are apparent benefits to residential humidification for control of influenza virus in the air and on surfaces and temporary relief from cough and cold symptoms, excessive indoor humidity can lead to unattended consequences such as mold and mildew growth [37]. This study shows that meaningful reductions of airborne influenza virus are possible at indoor moisture levels that are generally acceptable in terms of overall indoor air quality. However, the model demonstrated that the RH in the humidified bedroom can exceed 60%, an upper level limit recommended in widely accepted guidance manuals [38], especially in the radiant heat model. In most cases the time above 60% RH were short. However, care should be taken to follow humidifier manufacturer's guidance to ensure that moisture levels are maintained at acceptable levels. The risk of mold and mildew growth can be further reduced by raising the temperatures of surfaces through the addition of insulation or other means on condensing surfaces, such as windows and doors [39].

Our modeling effort focused on airborne influenza virus. One can be exposed to influenza by exposure to contaminated aerosols, large droplets, and direct contact with contaminated secretions or fomites. However, there is disagreement in the scientific community as to the relative importance of the various exposure routes [40-42]. A modeling study by Nicas and Jones, showed that the relative contribution of the airborne route of exposure may be modulated by the viral concentration in the salvia, with the higher the concentration the more likely that the airborne route is an important mechanism of exposure [43]. If the relative contribution of aerosol route of exposure is small, the impacts of humidification at minimizing airborne influenza survival may be similarly small. There is data, however, that indicates that increased humidification decreases survival of influenza virus on surfaces [40-42]. Therefore, while the focus of our modeling effort was on airborne influenza virus, humidification may reduce survival of the virus on household surfaces.

No studies were identified in which RH modeled by CONTAM or other models were used to address the impact of moisture levels on biological contaminants [44-46]. However, previous studies have employed CONTAM to assess humidity levels [45,46]. Using an earlier version of the CONTAM model, researchers from NIST compared model data with indoor RH measurements in two short time periods. While the data was preliminary, the model and measured results has a reasonable agreement [45], indicating the utility of CONTAM in modeling moisture levels.

This modeling study demonstrated differences in the impacts of humidification between forced air and radiant home heating methods. The median air exchange rate of the models was essentially the same, approximately 0.22 air changes per hours (ACH). However, due to lower air movement between rooms, radiant heat models showed larger increases in moisture levels, and therefore larger decreases in influenza virus. In a similar way, home characteristics such as the room volume and air exchange rate should be considered when evaluating the type and amount of humidification to be added to a home. ACH observed in our modeling effort are lower than previous modeling efforts primarily due to modeling only the winter months where windows are typically closed. Geographic areas should also be considered when interpreting these results. In areas with lower outdoor AH than the meteorological data we employed from Boston, MA, more humidification may be necessary to have a meaningful impact on indoor AH levels.

## Conclusions

Our results build upon previous efforts to evaluate the impacts of moisture on influenza virus survival [2-9,13,47]. These laboratory studies have consistently confirmed that survival of the influenza virus in the air and on surfaces is modulated by moisture levels, with the majority showing the lowest level of survival in the range of 40 to 60% RH. While field studies are necessary to confirm our modeling results, our findings suggest that indoor humidification will increase AH and RH to levels shown to reduce levels of the influenza virus. In this way, humidifiers may be an important tool to reduce survival influenza virus in the home. The effects of humidification on influenza virus survival, however, should be further evaluated with careful and controlled laboratory and field studies.

## Abbreviations

AH: absolute humidity; ACH: air changes per hours; mb: millibar; NIST: National Institute of Standards and Technology; RH: relative humidity; RT-qPCR: reverse transcription-quantitative polymerase chain reaction.

## Competing interests

The authors declare that they have no competing interests.

## Authors' contributions

MHK carried out the modeling analysis. JGA and DLM assisted in the design of the study and helped to draft the manuscript. MPF provided emissions information and helped to draft the manuscript. JJM participated in its design, provided emissions information and helped to draft the manuscript. TAM conceived of the study, and participated in its design and coordination and helped to draft the manuscript. All authors read and approved the final manuscript.
